# The relationship between self-esteem and procrastination among university students: the chain mediating effect of perfectionism and academic motivation

**DOI:** 10.3389/fpsyg.2026.1775527

**Published:** 2026-03-19

**Authors:** Chunmei Chen, Dandan Chen, Yujie Zhu, Fanghao Xiao, Yanlong Li, Xinxin Lin

**Affiliations:** 1 Teachers College, Jimei University, Xiamen, Fujian, China; 2 VNSAA St. Hilary’s School, Hong Kong, China; 3 Marxist College, Xiamen Institute of Technology, Xiamen, Fujian, China; 4 Department of Diplomacy and Foreign Affairs Management, China Foreign Affairs University, Beijing, China

**Keywords:** academic motivation, chain mediation, perfectionism, procrastination, self-esteem, university students

## Abstract

**Background:**

Procrastination among university students has adverse effects on their academic performance, daily life, and physical and mental health. Understanding the mechanisms underlying procrastination facilitates the development of effective interventions to alleviate its adverse impacts.

**Methods:**

To examine the effect of self-esteem on university students’ procrastination, and the chain mediating effects of perfectionism and academic motivation in this process, this study surveyed 2,523 undergraduates from five universities in Fujian Province using validated scales measuring self-esteem, perfectionism, academic motivation, and procrastination, and employed PROCESS Model 6 for statistical analysis.

**Results:**

(1) Self-esteem was significantly negatively correlated with procrastination (*β* = −0.200, *p* < 0.001); (2) perfectionism partially mediated the relationship between self-esteem and procrastination [95% CI = (0.032, 0.057), Boot SE = 0.007]; (3) academic motivation partially mediated the relationship between self-esteem and procrastination [95% CI = (−0.184, −0.135), Boot SE = 0.014]; (4) perfectionism and academic motivation served as joint sequential mediators in the effect of self-esteem on procrastination [95% CI = (−0.033, −0.019), Boot SE = 0.003].

**Conclusion:**

The findings of this study provide a theoretical basis for universities to design tailored interventions that enhance self-esteem, regulate maladaptive perfectionism, and strengthen academic motivation, thereby reducing procrastination and the associated risks of academic failure and psychological issues among university students.

## Introduction

Procrastination is a widespread and detrimental form of self-regulation failure. It is defined as a behavioral tendency wherein individuals voluntarily delay necessary tasks despite foreseeing the negative consequences of such delays. This phenomenon is characterized by voluntariness, avoidance, and irrationality (Tuckman, 1991; Lay, 1986; Van Eerde, 2000; Steel, 2007; Ferrari and Pychyl, 2012). It is a conscious behavior that delays the initiation or completion of tasks and results in psychological distress (Solomon and Rothblum, 1984). Procrastination undermines academic efficacy and their quality of life, posing a threat to physical and psychological health (Rabin et al., 2011; Klingsieck, 2013; Rahimi et al., 2023; Johansson et al., 2023). In severe instances, it may even precipitate maladaptive tendencies that predispose individuals to academic withdrawal and suicide (Lindner et al., 2023; Yang et al., 2025; Delgado et al., 2025). In China, procrastination is particularly prevalent among university students. Research indicates that students frequently exhibit this behavior at varying levels (Ren, 2019). Furthermore, studies have shown that moderately high levels of procrastination are prevalent among undergraduate students, which may induce negative emotions such as stress, anxiety, self-blame, and disappointment, thereby impeding academic development and mental health (Wu, 2023; He, 2023).

Given these consequences, procrastination among undergraduates has emerged as a critical concern for scholars worldwide. Investigating the mechanisms underlying procrastination among university students holds significant practical implications for effective prevention and intervention. Research indicates that self-esteem influences individuals’ beliefs regarding their ability to cope with challenges, thereby affecting their sense of self-regulatory efficacy (Al-Darmaki, 2012). A survey of 910 university students in Shandong Province, China, revealed that self-esteem significantly negatively predicts procrastination (Zhang, 2022). Studies have confirmed this negative correlation between self-esteem and procrastination (Tian, 2019; Wang, 2016). Similarly, a study involving 1,019 university students identified a positive correlation between perfectionistic high standards and procrastination (Tian, 2019). Additionally, some scholars have explored the relationship between academic motivation and procrastination among university students (Deng, 2012). Zhu and Ling (2016) conduct a study revealing a positive association between university students’ self-esteem levels and their implicit perfectionism—specifically, higher self-esteem is linked to stronger implicit perfectionism. Furthermore, Da (2015) investigate the interrelationships among university students’ self-esteem, self-determined academic motivation, and academic procrastination. In conclusion, existing literature suggests that university students’ procrastination is closely associated with self-esteem, perfectionism, and academic motivation, and the latter two may play a mediating role. Although substantial research has examined procrastination among university students, relatively few studies have provided in-depth and comprehensive analyses of its underlying mechanisms. The present study aims to address this gap.

### The relationship between self-esteem and procrastination

Self-esteem is an individual’s overall evaluation of their self- worth and capacity, which directly influences self-efficacy and emotional regulation (Zhang et al., 2018). James (1890) reckons that self-esteem is not fixed (James, 1890). Procrastination functions as a prophylactic buffer for self-esteem. Based on Lazarus and Folkman (1984) triple-A theory, individuals initially appraise a task when confronted with it. When the perceived task markedly exceeds one’s capabilities and self-esteem is low, the individual may fall into a psychological predicament characterized by self-denial, fear of failure, and reluctance to attempt the task. This predicament generates persistent anxiety. To avoid this anxiety, individuals ultimately resort to avoidance strategies as a way of self-protection, resulting in procrastination (Lazarus and Folkman, 1984). Numerous studies have indicated that self-esteem negatively predicts procrastination (Çapan, 2010; Zhang et al., 2018; Grunschel et al., 2018; Hu et al., 2018; Ferradás et al., 2020; Yang et al., 2021; Bodalski et al., 2023; Hidalgo-Fuentes et al., 2024; Zhuan et al., 2024). In summary, hypothesis 1 is proposed:

*H*1: Self-esteem negatively predicts procrastination.

### The mediating effect of perfectionism

Horney (1950) holds the view that perfectionists typically set unrealistically high standards and fixate on constructing an idealized self-image, conceptualizing perfectionism as a neurotic symptom. Its core characteristic manifests as a persistent preoccupation with self-perceived deficiencies and a compulsive drive to correct them, whereby individuals constantly seek out and attempt to conceal perceived imperfections (Horney, 1950). Extensive research indicates that perfectionists set exceptionally high standards for both themselves and others, exhibiting heightened sensitivity to their imperfections and shortcomings. They seek external validation and fear both negative appraisal and potential failure (Hollender, 1965; Burns, 1980; Frost et al., 1990). Ren (2019) conceptualizes perfectionism as an individual’ active and focused pursuit of a flawless ideal state, driven by high personal standards and stringent requirements. This pursuit is not pathological but rather a personality trait detectable in the general population. In the present study, perfectionism is characterized by setting high standards, striving to avoid failure, and pursuing excellence. On the one hand, self-esteem exerts a significant influence on perfectionism, and this is a fundamental psychological basis for understanding the developmental mechanism of perfectionism (Haley and Flannery-Schroeder, 2023). The level of self-esteem has a significant effect on how undergraduates cope with perfectionism tendencies, influencing their psychological stress and behavioral responses (Chufar and Pettijohn, 2013). In the pursuit of goals across academic, social, and other domains, individuals with high self-esteem are more inclined to endorse perfectionistic beliefs characterized by “the need to meet high standards” (Campbell and Paula, 2002). On the other hand, perfectionists tend to equate unattained high standards with failure. In such circumstances, procrastination serves as a psychological defense mechanism, enabling individuals to avoid the stress and negative emotion associated with potential failure. Consequently, perfectionists are more prone to procrastination (Ghosh and Roy, 2017; Çapan, 2010; Huang et al., 2023). In summary, Hypothesis 2 is proposed:

*H*2: Perfectionism mediates the relationship between self-esteem and procrastination.

### The mediating effect of academic motivation

Academic motivation is a critical psychological driver for students’ academic engagement, persistence, and effective time management (Wan et al., 2023). Self-esteem, as a key determinant of academic motivation among university students, serves as a reinforcing factor that sustains and enhances their drive to achieve (Moyano et al., 2020). High self-esteem enhances students’ enthusiasm and autonomy in learning, encouraging them to proactively engage in learning rather than passively receive knowledge (Komarraju and Dial, 2014). Students with high self-esteem demonstrate superior cognitive functioning and predictive abilities, manifesting a positive cognitive attitude toward academics, life planning, and coping with challenges, thereby strengthening their academic motivation. However, students with low self-esteem hold negative cognitions that attenuate the formation of academic motivation (Albagawi et al., 2025). Previous studies have revealed that that self-esteem positively predicts academic motivation (Komarraju and Dial, 2014; Komarraju and Dial, 2014). Furthermore, research has indicated that academic motivation serves as a crucial psychological resource that inhibits procrastination among university students (Elizondo et al., 2024). Students with weak academic motivation, often characterized by a lack of learning interest and an unclear sense of purpose, are particularly susceptible to procrastination (Lee, 2005; Abidin et al., 2023). Consequently, academic motivation acts as a significant negative predictor of procrastination (Yurtseven and Doğan, 2019; Koçer and Şeker, 2025; Wei and Mohd Noordin, 2025; Vlachopanou et al., 2025). In summary, Hypothesis 3 is proposed:

*H*3: Academic motivation mediates the relationship between self-esteem and procrastination.

### The chain mediating effect of perfectionism and academic motivation

A key motive for perfectionists is to cultivate a more favorable self-perception or self-image and to elicit specific responses or validation from others. A prominent external drive for perfectionists is their preoccupation with external evaluations and the attainment of externally defined standards. When these conditions are met, their learning motivation is further reinforced (Wan et al., 2023). Numerous studies have demonstrated that a greater inclination toward perfectionism among university students is associated with higher levels of academic motivation (Ghaemi and Damirchiloo, 2015; Xu, 2017; Chang et al., 2016; Mahasneh et al., 2019). Some scholars argue that perfectionists set high standards, are highly organized, and hold elevated expectations for themselves. They strive to achieve ambitious goals through diligent effort, as reflected in their strong academic engagement and active participation. They exhibit more pronounced intrinsic academic motivation (Stoeber and Rambow, 2007; Wang et al., 2007). Conversely, other scholars contend that perfectionists’ excessively high standards and fear of failure trigger anxiety, which may undermine academic motivation (Mills and Blankstein, 2000). Therefore, perfectionism can influence academic motivation through both adaptive and maladaptive pathways. In summary, Hypothesis 4 is proposed:

*H*4: Perfectionism and academic motivation serially mediate the relation between self-esteem and procrastination.

**Figure 1:**
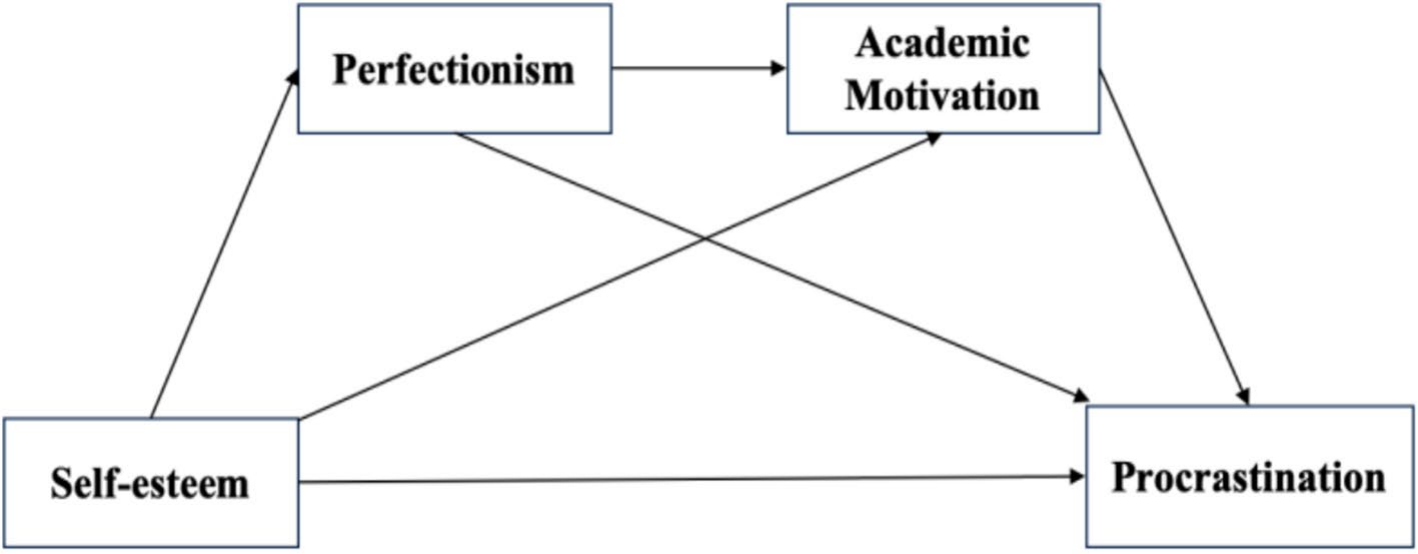
The theoretical serial mediation model.

The present study constructed a chain-mediation model to examine the relationship between self-esteem and procrastination among undergraduates, specifically investigating the joint indirect effects of perfectionism and academic motivation. The findings are expected to inform interventions by offering theoretical and practical insights aimed at enhancing students’ academic self-efficacy and reducing procrastination (Figure 1).

## Method

### Participants

The survey employed a convenience sampling method, recruiting participants from universities of varying tiers across different regions of Fujian Province, including Jimei University, Huaqiao University, Xiamen Institute of Technology, Quanzhou University of Information Engineering, and Fuzhou Technology and Business University. The questionnaire was distributed online via the Wenjuanxing platform, with instructors at each university assisting in organizing collective survey sessions during class breaks. Prior to participation, respondents were informed of the study’s purpose, confidentiality safeguards, voluntary nature, and their right to withdraw at any time, ensuring fully informed consent. After eliminating responses with excessively short completion times or obvious response patterns, 2,523 valid questionnaires retained.

The sample was relatively balanced across key demographic dimensions, including gender, major, and academic year. The gender distribution approached parity (51.13% male, 48.87% female). Regarding academic disciplines, engineering accounted for the largest proportion (33.02%), followed by humanities, sciences, medicine, agriculture, and other disciplines not specified. The distribution by academic year showed a monotonic decline from first-year (38.33%) to fourth-year students. In terms of geographical background, the majority of students came from urban areas (43.76%), followed by rural (29.65%) and township areas (26.60%). Overall, the sample structure was well-balanced, broadly reflecting the key characteristics of the university student population, and demonstrating good representativeness, making it appropriate for this research analysis (Table 1).

**Table 1:** Descriptive statistics of the sample.

Items	Category	Frequency	Percentage (%)
Gender	Male	1,290	51.13
	Female	1,233	48.87
Major	Humanities	554	21.96
	Sciences	438	17.36
	Engineering	833	33.02
	Medicine	196	7.77
	Agriculture	187	7.41
	Others	315	12.49
Year of study	Freshman	967	38.33
	Sophomore	565	22.39
	Junior	444	17.60
	Senior	301	11.93
	Others	246	9.75
Family residence	Urban	1,104	43.76
	Rural	748	29.65
	Township	671	26.60
Total		2,523	100.0

#### University Students’ Self-esteem Rating Scale

Self-esteem was measured using the Self-esteem Questionnaire for Undergraduates developed by Zhang and Song (2011). The scale consists of 18 items rated on a 5-point Likert scale and comprises four dimensions: sense of significance (e.g., “I always seek attention and affirmation from others”); sense of competence (e.g., “When encountering difficulties in learning or life, I always believe I can handle them properly”); sense of belonging (e.g., “I often actively participate in various activities organized by the school and department”); sense of appearance (e.g., “I think I am good-looking and dress well”). For each dimension, a higher average score indicates a higher level of self-esteem that specific aspect for the individual, whereas a lower average score reflects a lower level. After reverse-scoring the negatively worded items, an overall mean score was calculated, with higher overall mean score indicates a higher overall level of self-esteem for the individual. The Cronbach’s α coefficient for this questionnaire in this study was 0.705, and the KMO value was 0.848.

#### University Students’ General Perfectionism Scale

Perfectionism was assessed using the General Perfectionism Scale for university students, developed by Yang (2011). This scale comprises 14 Likert-type items rated on a 5-point scale and has demonstrated sound reliability and validity. Exploratory factor analysis reveal two common factors with eigenvalues exceeding 1, labeled “High Standards” and “Concern over Mistakes,” which together account for 51.61% of the cumulative variance explained (Yang, 2011). Sample items for the High Standards dimension include “I must spare no effort to achieve my goals,” “When competing with others, I always strive to be the best,” and “I expect to outperform most people.” Sample items for the Concern over Mistakes dimension include “Finding a single mistake in my studies/work drives me crazy,” “Making an error makes me feel inferior,” and “Even when I try my best, I still feel dissatisfied.” Each dimension was scored by computing the mean of its constituent items, with higher scores indicating stronger perfectionism. The Cronbach’s α coefficient for this questionnaire in this study was 0.645, and the KMO value was 0.795.

#### University Students’ Academic Motivation Scale

Academic motivation was assessed using the University Students’ Academic Motivation Questionnaire developed by Tian and Pan (2010), which has demonstrated sound reliability and validity. This 34-item scale employs a 5-point Likert-type response format and encompasses four distinct dimensions. The epistemic curiosity subscale includes items such as “I consistently derive enjoyment from university studies” and “My academic interest in my major discipline intensifies with continued study progression.” The ability pursuit subscale includes items such as “I often remind myself to continuously improve my analytical and problem-solving skills during the learning process” and “I study hard so that I can achieve something significant in the future.” The reputation pursuit subscale includes “I want to improve my standing in the class through hard work” and “I always want to earn the respect of others by improving my academic performance.” The altruistic orientation subscale includes “I often think that if I do not study diligently,” “I will be letting down my teachers” and “I really want to use my talents to contribute to my hometown.” The first two dimensions reflect intrinsic motivation, while the latter two represent extrinsic motivation. An overall mean score was computed across all items, with a higher average indicating stronger academic motivation. The Cronbach’sαcoefficient for this questionnaire in this study was 0.841, and the KMO value was 0.919.

#### University Students’ Procrastination Scale

The Aitken Procrastination Inventory (API), originally developed by Aitken (1982) and later revised for Chinese university students by Chen et al. (2010), is a 19-item self-report instrument designed to assess chronic academic procrastination. The scale has demonstrated sound reliability and validity through analysis of internal consistency, criterion-related validity, and empirical validity (Chen et al., 2010). Items are rated on a 5-point Likert scale, with 9 items reverse-scored. Typical positively worded items include “I always wait until the last minute to start things,” “Even if something has to be done, I do not start on it right away,” and “I usually begin tasks too late to finish them on time.” Representative reverse-scored items include “I make sure library books are returned on time,” “I consistently complete daily assignments according to the required schedule,” and “When I feel a task must be done, I do not put it off.” After reverse-scoring the relevant items, an average score was computed across all 19 items, with higher scores indicating greater procrastination severity. The Cronbach’sαcoefficient for this questionnaire in this study was 0.704, and the KMO value was 0.838.

#### Research design and data processing

To ensure data validity and the reliability of subsequent findings, the analyses proceeded according to the following statistical procedures. First, SPSS 26.0 was employed to conduct descriptive statistics for all key variables, including means, standard deviations, and other basic indicators. In addition, Pearson correlation analysis was used to examine the linear relationships among variables. Subsequently, multicollinearity was diagnosed using the variance inflation factor (VIF); any variable with a VIF exceeding the threshold of 10 was regarded as having serious collinearity and was consequently removed from the analysis. After confirming the absence of severe multicollinearity, we utilized Model 6 in the PROCESS macro to test the serial-mediation path (Hayes, 2017). Furthermore, to accurately estimate the significance of the mediating effects, the bias-corrected nonparametric percentile Bootstrap method was employed for resampling. A mediating effect was considered statistically significant if its 95% confidence interval did not include zero (Erceg-Hurn and Mirosevich, 2008). Finally, to assess potential common method bias, Harman’s single-factor test was performed before the main analysis to ensure that systematic method variance does not substantially distort the findings (Podsakoff et al., 2003).

## Research results

### Common method bias test

Data collected via self-report methods are potentially susceptible to common method bias. To address this concern, Harman’s single-factor test was conducted in the present study. The results revealed 23 factors with eigenvalues greater than 1, and the first unrotated factor explained 9.919% of the total variance—well below the critical threshold of 40%. Consequently, no serious common method bias was identified.

### Descriptive statistics and correlation analysis

Table 2 presents the means, standard deviations, and Pearson correlation coefficients among the four key variables: procrastination, perfectionism, self-esteem, and academic motivation. All correlations reached statistically significant levels, supporting the feasibility of subsequent mediation analyses.

**Table 2:** Descriptive statistics and correlation matrix for each variable.

Items	Mean	Standard deviation	Perfectionism	Self-esteem	Academic motivation	Procrastination
Perfectionism	3.072	0.519	1			
Self-esteem	3.108	0.488	0.301**	1		
Academic Motivation	3.183	0.485	0.338**	0.479**	1	
Procrastination	2.761	0.508	−0.005	−0.192**	−0.368**	1

**p < 0.01.

### Relationship between self-esteem and procrastination: a chain mediation model

**Figure 2:**
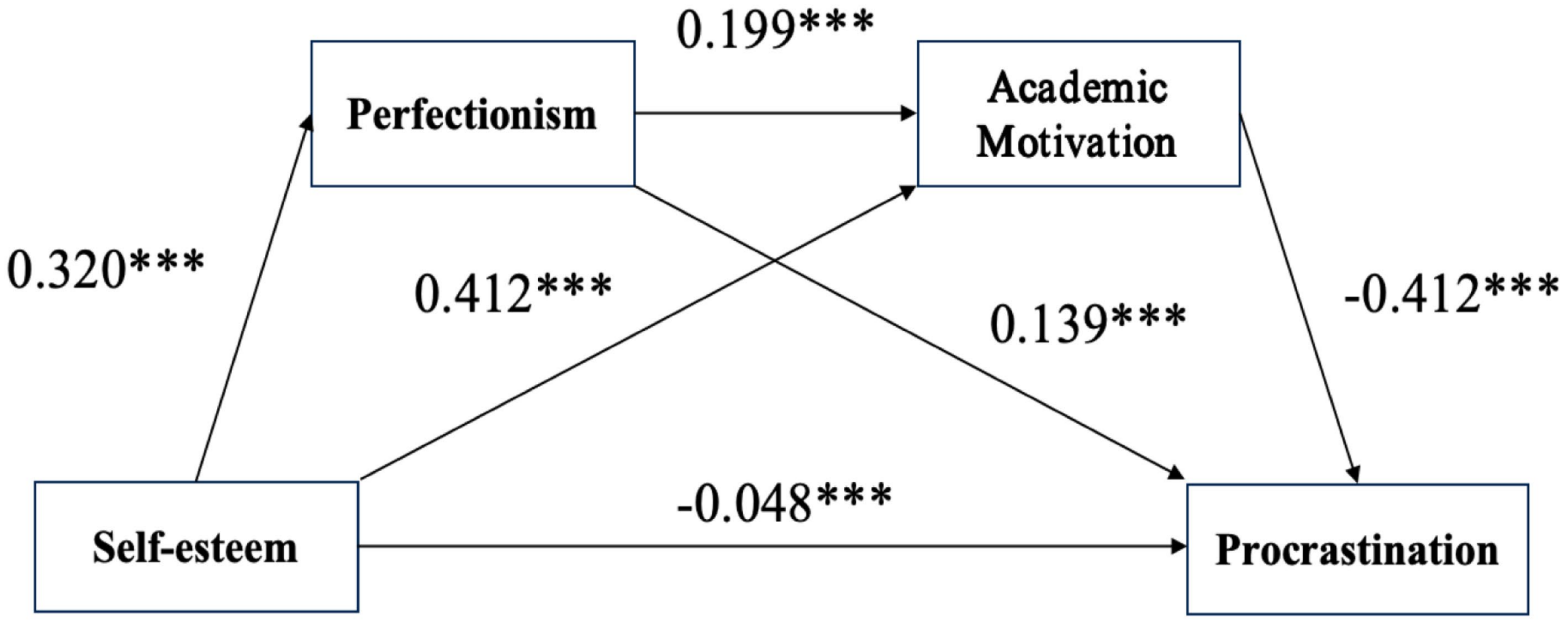
The chain mediation model. *p < 0.05; **p < 0.01; **p < 0.001.

The correlation analysis indicated significant associations among the variables, raising the possibility of multicollinearity. Therefore, before testing the mediation effects, the predictor variables in the equations were standardized and subjected to multicollinearity diagnostics. The results showed that the variance inflation factors (VIFs) for all predictors (1.200, 1.243, and 1.125) were all below the recommended threshold of 5. These results confirm the absence of severe multicollinearity, indicating that the data are suitable for subsequent mediation analysis. The chain mediation model was tested using Hayes’s Process Macro (Model 6) with 5,000 bootstrap samples to estimate the 95% confidence intervals (CI) for the mediating effects of perfectionism and academic motivation. The results are shown in Figure 2 and Table 3.

**Table 3:** The regression equation of chain mediation.

Items	Perfectionism		Academic motivation		Procrastination		Procrastination	
	β	t	β	t	β	t	β	t
Constant	2.079***	32.724	1.292***	20.355	3.383***	52.885	3.797***	49.119
Self-esteem	0.320***	15.828	0.412***	23.252	−0.200***	−9.837	−0.048***	−2.197
Perfectionism			0.199***	11.932			0.139***	7.193
Academic Motivation							−0.412***	−18.334
$R^{2}$	0.09		0.270		0.037		0.153	
Adjusted $R^{2}$	0.09		0.270		0.037		0.152	
F	250.54		467.146		96.762		151.767	

***p < 0.001.

The results showed that self-esteem was significantly negatively correlated with procrastination (*β* = −0.200, *p* < 0.001). After including perfectionism and academic motivation as mediators, self-esteem remained significantly negatively correlated with procrastination, although the effect size was notably reduced (*β* = −0.048, *p* < 0.001). Furthermore, self-esteem was significantly positively correlated with perfectionism (*β* = 0.320, *p* < 0.001), and both self-esteem and perfectionism were significantly positively correlated with academic motivation (*β* = 0.412, *p* < 0.001; *β* = 0.139, *p* < 0.001, respectively). Finally, academic motivation significantly negatively correlated with procrastination (*β* = −0.412, *p* < 0.001).

Further analysis of the mediation effects (see Table 4) revealed that the total indirect effect of perfectionism and academic motivation was significant, as the Bootstrap 95% CI did not include zero. This indicates that perfectionism and academic motivation jointly mediate the relationship between self-esteem and procrastination. The total effect of self-esteem on procrastination was −0.200, with a direct effect of −0.048 accounting for 24% of the total effect and a total indirect effect of −0.152 accounting for 76% of the total effect. This mediation effect was manifested through three distinct pathways:

1. Self-esteem → Perfectionism → Procrastination: The indirect effect was 0.044 [95% CI = (0.032, 0.057), Boot SE = 0.007], supporting H2.
2. Self-esteem → Academic Motivation → Procrastination: The indirect effect was −0.170 [95% CI = (−0.184, −0.135), Boot SE = 0.014], supporting H3.
3. Self-esteem → Perfectionism → Academic Motivation → Procrastination: The chain mediation effect was −0.026 [95% CI = (−0.033, −0.019), Boot SE = 0.003], supporting H4.

**Table 4:** Bootstrap analysis of the mediation effect test.

Items	Effect	Boot SE/SE	Boot LL CI	Boot UL CI	Relative mediation effect
Total	−0.200	0.020	-	-	
Direct effect Self-esteem→Procrastination	−0.048	0.022	-	-	24%
Total Indirect effect	−0.152	0.015	−0.171	−0.112	76%
Self-esteem→Perfectionism→Procrastination	0.044	0.007	0.032	0.057	-
Self-esteem→Academic Motivation→Procrastination	−0.170	0.014	−0.184	−0.135	-
Self-esteem→Perfectionism→Academic Motivation→Procrastination	−0.026	0.003	−0.033	−0.019	-

## Discussion

### The effect of self-esteem on procrastination

The results of this study indicated that self-esteem was negatively correlated with procrastination. Specifically, university students with higher self-esteem exhibited lower levels of procrastination, whereas those with lower self-esteem showed higher levels of procrastination. This conclusion aligned with the majority of extensive studies. When university students have high self-esteem, their confidence and sense of control over task completion are stronger, positively influencing task performance. Conversely, when university students have low self-esteem, their perceived control and confidence in completing tasks are diminished, making them more likely to adopt avoidance behaviors to mitigate the experience of failure, ultimately leading to procrastination. This behavior, in turn, exerts a deleterious effect on academic achievement, and poor performance subsequently reinforces this tendency (Raza et al., 2020; Ghasempour et al., 2024). Many studies have indicated that improved self-esteem contributes to greater self-efficacy and emotional regulation, thereby reducing procrastination in both daily life and academic contexts. Students with higher self-esteem tend to exhibit greater emotional stability, a stronger sense of competence and satisfaction, as well as greater persistence and coping capacity when facing challenging tasks. They are less likely to resort to avoidance behaviors, significantly reducing the risk of procrastination. In contrast, university students with lower self-esteem, in an effort to protect their self-esteem and avoid feelings of failure, are more prone to engaging in procrastination (Duru and Balkis, 2017; Awwad et al., 2022; Acosta-Gonzaga, 2023; Theobald et al., 2023). Self-doubt and fear that failure will expose inadequacies prompt these students to delay tasks, a pattern consistent with the conceptualization of procrastination as a form of self-protection (Araya-Castillo et al., 2023).

Self-esteem serves as a psychological foundation for procrastination (Klassen et al., 2008; Al-Darmaki, 2012; Zhang et al., 2018). Mechanistically, procrastination is often employed by university students as a self-handicapping strategy, with its core motivation being the protection of self-esteem (Barutçu Yıldırım and Demir, 2020). It also serves as a maladaptive emotion-regulation strategy by which students temporarily escape task-related distress (Adu, 2014). From the perspective of Bandura (1977) self-efficacy theory, the relation between self-esteem and procrastination can be interpreted through the lens of distorted capability appraisals, which constitute a maladaptive cognitive substrate for procrastinatory behavior (Bandura, 1977). Students with low self-esteem typically hold negative perceptions regarding their self-worth and sense of competence. The underlying intense fear of failure leads individuals to avoid goals actively, diminishes their need for autonomy, and increases the risk of problematic procrastination in both academic and daily life contexts (Haghbin et al., 2012; Martinie et al., 2022; Yang et al., 2025). Although a few studies have found no direct effect of self-esteem on procrastination, suggesting instead an indirect influence through time management behaviors (Han et al., 2014). However, the majority of research has documented a negative association between self-esteem and procrastination. Therefore, families and universities should pay close attention to students’ psychological states, focus on their intrinsic worth, and provide sustained trust and care to elevate their self-esteem.

### The mediating role of perfectionism

This study revealed that perfectionism partially mediates the relationship between self-esteem and procrastination. Specifically, higher self-esteem predicted elevated perfectionism, which in turn increased procrastination among university students. The level of self-esteem appears to shape the cognitive appraisal and emotion-regulation processes underlying the perfectionistic standard, ultimately affecting their procrastinatory behavior. In this context, procrastination acts as a defensive mechanism that temporarily alleviates the psychological discomfort caused by high standards and self-criticism (Chang, 2014). Previous research has confirmed that university students’ self-esteem was positively correlated with their perfectionism. Individuals who hold positive views of their own abilities are more inclined to set stringent performance goals as a means of validating their self-worth, which can foster perfectionism (Abouserie, 1995; Chufar and Pettijohn, 2013). Consistent with this perspective, these students maintain elevated self-evaluations and strive to preserve and enhance a positive image by meeting demanding standards (Al-Darmaki, 2012; Dunkley et al., 2012; Borji et al., 2020; Haley and Flannery-Schroeder, 2023).

From the perspective of self-determination theory, individuals with high self-esteem, who often experience sufficient satisfaction of psychological needs such as self-worth, autonomy, and relatedness, tend to set high standards for themselves. This represents a form of positive perfectionism associated with the fulfillment of higher psychological needs (Miquelon et al., 2005). At the mechanistic level, individuals with high self-esteem possess a secure and stable self-identity, rendering them less susceptible to self-doubt in the face of short-term difficulties. Accordingly, their perfectionistic striving manifests as proactive task mastery rather than fear-driven avoidance (Singh and Bala, 2020). Furthermore, numerous studies have confirmed that university students’ perfectionism was positively correlated with procrastination. Flett et al. (1992) noted that when university students perceive high external standards and strong social pressure, they are more likely to exhibit procrastination. Fear of failure has been identified as one of the most significant contributing factors on procrastination (Flett et al., 1992; Sirois et al., 2017). The psychological stress arising from perfectionism undermines students’ task initiation (Coutinho et al., 2022). Perfectionistic individuals tend to equate errors with failure and are excessively concerned about others’ negative evaluations, leading them to adopt procrastination as a defensive strategy to avert potential failure (Shih, 2017). Perfectionism denotes a cognitive-behavioral constellation defined by the pursuit of flawlessness, meticulous attention to detail, and the aspiration for excellence. Individuals high in perfectionism often routinely allocate disproportionate time and cognitive resources to deliberating on the optimal manner of task execution, repeatedly engaging in anticipatory rumination that delays the implementation and progressively intensifies procrastination (He, 2023). The underlying mechanisms can be explained theoretically from two perspectives: First, based on the appraisal-anxiety theory, perfectionists tend to set high standards for tasks, which often induces anxiety. If they perceive that these standards cannot be met, they may resort to procrastination as a means to avoid the “threat of failure”. Second, self-regulation perspectives highlight that perfectionists are often caught in an intrapsychic conflict between elevated goal expectations and diminished self-efficacy. They perceive a significant gap between their current state and their ideal goals, making it difficult to initiate tasks and leading to procrastination (Sirois et al., 2017; Ashraf et al., 2023). Taken together, these findings underscore the need for families, universities, and relevant educational authorities to pay greater attention to perfectionism among university students.

### The mediation effect of academic motivation

The present findings revealed that academic motivation partially mediated the relationship between self-esteem and procrastination. That was, augmented sense of self-esteem was associated with enhanced academic motivation, which in turn attenuated the propensity to procrastinate. This mediational pathway converges with prior evidence indicating that self-esteem embodies a pivotal psychological resource that fuels students’ learning behaviors. At the mechanistic level, research has shown that self-esteem cultivates academic motivation by strengthening students’ sense of self-worth and fostering firm confidence, thereby facilitating more active and agentic engagement in academic activities (Abouserie, 1995; Takahashi and Takahashi, 2013). University students with high self-esteem have positive and constructive cognitive frameworks, which results in lower levels of anxiety and negative emotions during the learning process. They typically exhibit stronger academic motivation and less procrastination (Carranza Esteban et al., 2022). Such students are more likely to believe that sustained effort can lead to self-improvement and hold positive expectations toward academic tasks, thereby demonstrating greater initiative and persistence. In contrast, students with low self-esteem are more prone to self-doubt and insecurity, which can undermine their academic motivation and even lead to avoidant behaviors (Basco and Han, 2016; Acosta-Gonzaga, 2023). They are more prone to developing cognitive distortions that trigger negative emotions, leading to emotional detachment from academic tasks and behavioral avoidance. This form of disengagement indirectly contributes to a decline in academic performance, establishing a detrimental cycle of procrastination (Ratanasiripong et al., 2021).

Academic motivation has become a significant psychological factor influencing procrastination among university students. Students with strong academic motivation are more inclined to proactively manage their time, plan tasks effectively, and demonstrate a higher degree of self-regulation, enabling them to overcome procrastination effectively (Abidin et al., 2023). Elizondo et al. (2024) found that academically motivated students display enhanced task immersion and deliberate implementation intentions, utilizing adaptive learning tactics including temporal regulation and metacognitive monitoring, which fosters sustained persistence and thereby mitigates procrastination. On the contrary, students with motivational deficits exhibit an impoverished self-regulatory repertoire; often compelled by avoidance motivation or failure-anticipation schemata, they reflect pronounced procrastinatory inclinations. Furthermore, their study distinguished between active procrastination and passive procrastination. They argue that highly motivated students may sometimes engage in active procrastination, consciously delaying tasks to achieve a better state for completion. Students with low motivation often exhibit passive procrastination accompanied by negative emotions and executive dysfunction (Elizondo et al., 2024). Although the present study did not differentiate between active and passive procrastination, the findings confirmed that academic motivation was negatively correlated with procrastination and thus served as a significant negative predictor. Furthermore, the results revealed the partial mediating role of academic motivation in the relationship between self-esteem and procrastination.

### The chain mediating effect of perfectionism and academic motivation

The present study identified a close coupling between perfectionism and academic motivation, which jointly function as sequential mediators in the pathway from self-esteem and procrastination: self-esteem → perfectionism → academic motivation → procrastination. Specifically, undergraduates with higher self-esteem reported stronger perfectionism, which subsequently enhanced their academic motivation and ultimately reduces procrastinatory behavior. University students with higher self-esteem commonly illustrate a more stable sense of self-worth and a clearer self-concept. This positive self-representation motivates them to set higher performance standards for themselves, leading to the adoption of more rigorous criteria in goal-setting (Miegel et al., 2020; Borji et al., 2020; Lipińska-Grobelny and Bednarek, 2021). Undergraduates of this profile typically exemplify adaptive and rational perfectionism, that is, while adhering to rigorous standards, they demonstrate a generous acknowledgment of personal fallibility and refrain from inordinate self-reproach, thereby transmuting perfectionistic strivings into a driver of self-actualization rather than a precursor to procrastination or avoidance behaviors (Chufar and Pettijohn, 2013).

Numerous studies have confirmed that university students’ perfectionism is positively correlated with academic motivation, a finding consistent with the conclusions of this research. Perfectionists’ pursuit of high standards can translate into effective motivational drive, prompting university students to initiate tasks promptly and accomplish their goals efficiently (Athulya et al., 2016). Such students are more inclined to adopt effective learning strategies and resource management approaches, actively investing effort to meet their self-imposed high standards and expectations (Stoeber and Rambow, 2007). Burnam et al. (2014) pointed out that perfectionists tend to set high standards, impose strict self-demands, and emphasize organization and self-improvement. This form of perfectionism fosters positive academic motivation, such as intrinsic motivation and mastery goal orientation, which helps students actively regulate their learning behaviors and reduce procrastination (Burnam et al., 2014).

However, several discrepant findings emerged. Bong et al. (2014) posit that perfectionists, influenced by inordinate external pressures and excessive social expectations, tend to adopt performance goal orientations-specifically performance-approach and performance-avoidance goals—which render their academic motivation susceptible to the adverse effects of anxiety and stress, thereby increasing the propensity for procrastination (Burnam et al., 2014). These findings suggest that the influence of perfectionism on academic motivation is not deterministic; rather, efforts should be directed toward harnessing and enhancing the positive aspects of individuals’ perfectionism.

## Contribution, limitations and prospects

### Contributions

This study examines the pathway of self-esteem → perfectionism → academic motivation → procrastination. The findings indicate that self-esteem can reduce procrastination through the chain-mediating effect of perfectionism and academic motivation. However, it is important to note that the mediating path self-esteem → perfectionism → procrastination may increase procrastination. In contrast, the path self-esteem → academic motivation → procrastination tends to decrease procrastination. This study further clarifies the mediating roles of perfectionism and academic motivation in the relationship between self-esteem and procrastination. It contributes to a deeper and more comprehensive understanding of the chain-mediating effects of perfectionism and academic motivation in the link between self-esteem and procrastination, offering a new perspective for interpreting the multi-factor mechanisms underlying procrastination among university students. These insights can inform targeted intervention strategies in higher education to address student procrastination and mitigate its negative impacts. The following implications can be drawn from this research.

Firstly, to enhance university students’ self-esteem, higher-education institutions need to prioritize its development through psychological guidance and instructional interventions, thereby fostering greater self-acceptance and reducing intrapsychic conflict (Chufar and Pettijohn, 2013). Educators can offer low-stakes experiential platforms, such as club activities, volunteer initiatives, and micro-competitions, that enable iterative enactive mastery, incrementally strengthening self-regulatory efficacy while attenuating fear of failure. Counselors to conduct systematic individual consultations, disseminate evidence-based information on self-acceptance, and institutionalize sustained psychosocial support mechanisms that foster perceived self-worth. Furthermore, institutions are morally and pedagogically obliged to implement a multi-dimensional assessment regime capable of delivering domain-specific, positive feedback to learners with diverse strengths, thereby elevating global self-esteem and, concomitantly, reducing academic procrastination.

Secondly, the adaptive channeling of perfectionism requires a collaborative effort from a synergistic educational-support system involving universities, instructors, parents, and the students themselves. Universities should guide students to internalize ideals, convictions, and long-term aspirations, inspiring them to pursue sustained excellence and ascend to intellectual growth. In practice, universities may implement case-based seminars to help students recognize that error is an inherent part of development, thereby fostering a cognitive reorientation from “maximalist perfectionism” to “incremental self-refinement” and reducing the affective burden associated with imperfection or failure. Furthermore, universities should refine their teaching and evaluation mechanisms by incorporating students’ ongoing performance and progress into assessment criteria. This helps create an inclusive learning environment that enhances students’ tolerance for errors and supports the development of a healthier self-concept. Educators should assist students in forming realistic self-assessments, setting appropriate goals, and developing a clear understanding of their own abilities. They need to motivate students to strive diligently and pursue continuous self-improvement. Parents, as the primary support system at home, should provide ample emotional and social backing, offering consistent affirmation and encouragement to foster their children’s growth and development holistically. On an individual level, students are advised to define clear objectives for various stages of their academic journey and commit to continuous self-improvement. Ultimately, when university students successfully channel their perfectionism toward becoming better versions of themselves, their propensity for procrastination diminishes accordingly.

Thirdly, universities should assist students in clarifying the purpose of learning by integrating course content with personal growth and societal needs. For example, professional internships and applied projects allow students to experience the practical value of knowledge, thereby stimulating intrinsic motivation. Institutions should also create immersive learning environments, such as dedicated library study zones, laboratory open days, and activities like academic salons and discipline-based competitions—to foster a positive and motivated group atmosphere. Faculty members should focus on cultivating students’ interest in learning. Instructors can introduce interactive formats such as case-based teaching and project-based learning, allowing students to discover the appeal of knowledge through exploration. Meanwhile, teachers should guide students in setting step-by-step goals, breaking down larger tasks into weekly plans and daily checklists, allowing a sense of accomplishment to accumulate through the completion of smaller objectives. Peer support is also crucial; forming study groups can create positive peer modeling and healthy competition. Faculty are further advised to operationalize egalitarian communication channels that afford timely diagnosis of students’ academic impediments and deliver requisite psychosocial support. Concomitant enhancements in students’ motivational regulation are expected to significantly ameliorate procrastinatory behaviors. It is therefore posited that strengthening university students’ motivational regulation will significantly attenuate the occurrence of procrastination.

### Limitations and prospects

This study still has several limitations. First, the use of cross-sectional data limits the representativeness of the sample and constrains the ability to draw robust causal inferences regarding the relationships between variables. Second, the convenience sampling method may introduce selection bias, and the research did not conduct differential analyses of the mechanisms influencing procrastination across demographic variables such as gender, major, academic year, hometown type, or institution type, which may affect the accuracy and comprehensiveness of the findings. Third, due to time and resources, constraints the study did not classify and examine the subdimensions of perfectionism, academic motivation, and procrastination, which may restrict the depth of the conclusions. Finally, Harman’s single-factor test alone is insufficient to rule out common method bias in self-report research. In light of these constraints, future research should incorporate diverse data collection methods and prioritize longitudinal tracking studies for verifying causal relationships between variables. Subsequent work could use long-term follow-up data to analyze differences in the mechanisms of procrastination across relevant demographic variables, and to explore correlations among the subdimensions of perfectionism, academic motivation, and procrastination, thereby strengthening the reliability of the findings.

## Data Availability

The original contributions presented in the study are included in the article/supplementary material, further inquiries can be directed to the corresponding author.
